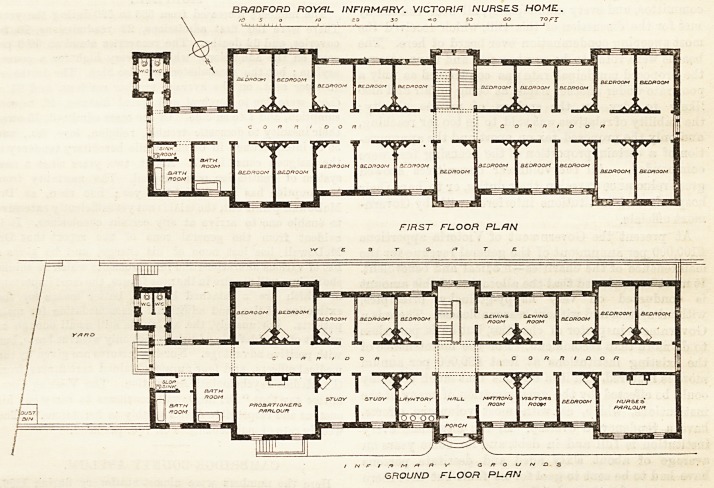# Hospital Construction

**Published:** 1898-02-12

**Authors:** 


### HOSPITAL CONSTRUCTION.

### BRADFORD ROYAL INFIRMARY.—VICTORIA NURSES' HOME.

The foundation-stone of this building was laid last August by the Duke of Devonshire, and the building is now approaching completion. It consists of a basement, which is only below ground on one side, and three upper floors, and stands in the grounds of the infirmary, with its fronts facing north-east and south-west respectively. The basement contains the caretaker's rooms (living-room, two bedrooms, scullery, &c., separately approached), a kitchen for the servants, a larder, linen-rooms, box-rooms, a boiler-room, and four spare rooms. In the centre of the ground floor is the main entrance, facing the infirmary, and leading to a central corridor, 7½ ft. wide, which runs the whole length of the building, and is lighted by large windows at each end, and by the staircase windows in the centre. The rooms on the left and right of the entrance are respectively the probationers'-room, two studies, a lavatory, matron's-room, visitors'-room, a bedroom, and the nurses' parlour, the two largest rooms being at each end. The lavatory and cloak-room is well placed close to the entrance, and there are two bath-rooms and a slop-sink at one end of the corridor, with two w.c.'s. opposite them, properly disconnected by a cross-ventilated passage. The opposite side of the corridor is occupied by bedrooms, each with a fireplace, and varying from 15 ft. by 10 ft. to 12 ft. by 10 ft., and 12 ft. high.

The first and second floors are repetitions of those below, but bedrooms occupy both sides of the corridor, and on the upper floor is a sick-ward for three beds, making, with the 49 bedrooms, accommodation for 52 inmates. The whole building is heated with hot water, the fireplaces being merely auxiliary. The baths and w.c.'s are one-eighth of the beds, and the slop-sinks one-seventeenth, which is quite sufficient, without being extravagant, and the general arrangement of the building seems to leave little to be desired. The only criticisms we would make are that the bedrooms might have been somewhat smaller; that 12 ft. is an unnecessary height for the ground floor; and that it would have been better to have placed the sitting-rooms on the south-west front, where they would have had plenty of sun, instead of on the north-east, but there may be special reasons for the position they are shown to occupy. The cost has been about £8,000, or £160 per bed (without furnishing), and the architects are Messrs. Milnes and France, of Bradford.

## Figures and Tables

**Figure f1:**